# Embolic ST-elevation Myocardial Infarction from Candida Endocarditis

**DOI:** 10.7759/cureus.7833

**Published:** 2020-04-25

**Authors:** Amre Ghazzal, Gauravpal S Gill, Sohab Radwan, Christopher Barnett

**Affiliations:** 1 Internal Medicine, MedStar Washington Hospital Center, Washington, USA; 2 Cardiology, MedStar Washington Hospital Center, Washington, USA

**Keywords:** embolic myocardial infarction, infective endocarditis, candida endocarditis, candida albicans

## Abstract

Infective endocarditis in intravenous drug users is uncommon in left-sided native valves. Adding to the rarity, in this case, is endocarditis from Candida species complicated by ST-elevation myocardial infarction. Embolic myocardial infarction has worse outcomes as compared to other etiologies, and the management of septic embolic myocardial infarction is rather challenging. The management of embolic myocardial infarction from Candida endocarditis vegetation includes antifungal therapy. The use of anti-thrombotic therapy and anticoagulation carries a significant risk of fatal neurologic complications and has been controversial, with limited observational data available. Among percutaneous coronary interventions, balloon angioplasty and stenting have been associated with multiple complications while aspiration embolectomy appears to be a safer option. Surgical management is considered if medical and interventional therapies fail or if there is an indication for valve replacement.

## Introduction

Acute coronary syndrome (ACS) describes a range of myocardial ischemic states that includes unstable angina and non-ST and ST-segment elevation myocardial infarction. It is associated with substantial morbidity and mortality and places a large financial burden on the healthcare system [1].

Causes of ACS include plaque rupture, coronary ectasia with thrombosis, coronary artery dissection, vasospasm, and embolism. Coronary embolism can appear in several contexts, including cardiomyopathy, rheumatic heart disease, left ventricular aneurysm, atrial fibrillation, prosthetic valve thrombosis, atrial myxoma, and infective endocarditis [2].

We describe a rare case of embolic myocardial infarction in an intravenous (IV) drug user who presented with aortic valve endocarditis from Candida infection.

## Case presentation

A 38-year-old male with active IV drug use presented to an outside hospital with fever, episodic flushing, and swelling in his right hand and forearm. Workup at the outside hospital included blood cultures positive for unspeciated yeast and a transthoracic echocardiogram demonstrating a normal ejection fraction (65%) and a 1.9 cm in diameter vegetation attached to the right coronary cusp of the aortic valve. The patient was transferred to our hospital in anticipation of aortic valve replacement.

**Figure 1:**
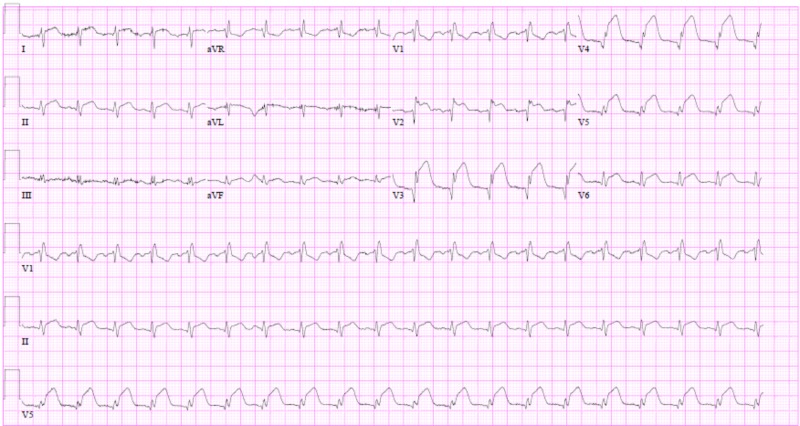
Electrocardiogram depicting a sinus rhythm, PR interval of 176 ms, right bundle-branch block, and ST elevations in the anterior and lateral leads

**Figure 2:**
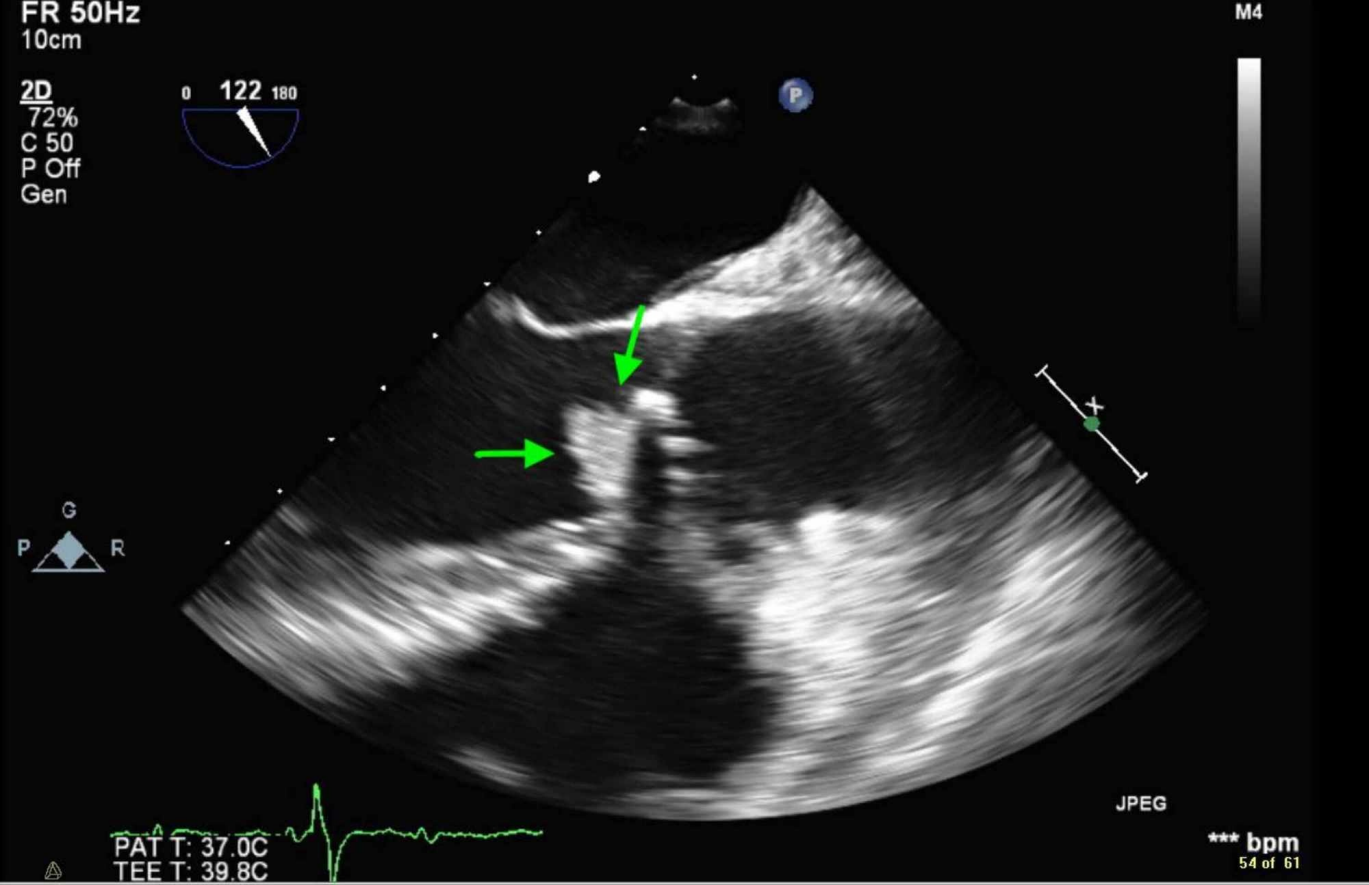
Transesophageal echocardiogram showing a 9x9 mm echodensity attached to the ventricular side of the aortic valve, which represents vegetation

While awaiting surgical intervention, the patient acutely developed sub-sternal chest pain. An electrocardiogram was acquired, which showed normal sinus rhythm, a PR interval of 176 ms, ST-segment elevations in the anterior and lateral leads (Figure 1), and a troponin-I elevation to 73.4 ng/ml (normal range <0.045 ng/ml). A transthoracic echocardiogram was performed and revealed a severely reduced ejection fraction of 25%-30%. The apex, apical wall of the septum, and the anterior wall were severely hypokinetic. The aortic valve vegetation was 9 mm in diameter, notably smaller as compared to the prior study (Figure 2).

On visual inspection, an abscess of the aortic valve and root extending into the interventricular septum was noted. Extensive debridement of the annular abscess with bovine pericardium patch repair and aortic valve replacement with a 23 mm bioprosthetic valve were performed. A complete heart block was subsequently diagnosed and a permanent pacemaker was placed. The patient was discharged to a skilled nursing facility for the remainder of a six-week micafungin therapy. On completion of parenteral antifungal therapy, lifelong oral fluconazole was initiated.

## Discussion

Acute coronary syndromes in patients with infective endocarditis are associated with a higher incidence of heart failure, cardiogenic shock, complete atrioventricular block, and mortality [3]. The case described in this report depicts the importance of early intervention, especially with fungal endocarditis. We report an extremely rare case where native aortic valve endocarditis from Candida spp. causes embolic acute coronary syndrome [4].

The most commonly reported mechanism for myocardial ischemia in patients with Candida endocarditis is coronary artery compression from a peri-annular abscess and pseudoaneurysm formation, while, infrequently, it can be caused by an embolic infarct [5].

Coronary embolism (CE) accounts for 2.9% of acute coronary syndrome and 4% of CEs are due to infective endocarditis [6]. In several studies, the left anterior descending (LAD) artery was reported to be the most commonly affected vessel and the majority of embolic myocardial infarctions occur in the setting of aortic valve endocarditis as compared to the mitral valve [3,5]. However, in another study, the incidence of the embolic acute coronary syndrome was similar in the right coronary artery and left circumflex and left anterior descending artery territories. The higher incidence reported in left coronaries distribution, namely, the left anterior descending, was explained by bias due to the fact that arteries with larger territories are more likely to be involved in autopsy cases [6].

Fungal endocarditis accounts for <2% of infective endocarditis cases [7-8]. Candida albicans was identified in 24% of fungal endocarditis cases [6]. It is more common in patients who are immunocompromised, post-cardiac surgery, have prosthetic valves, or indwelling catheters [6-8]. Early detection and intervention in these cases is important, as fungal infective endocarditis has been reported to have higher mortality as compared to non-fungal organisms [7].

Treatment involves antimicrobial therapy with intravenous anti-fungal agents followed by long-term suppressive oral anti-fungal agent use due to a high risk of recurrence. Data on the use of antithrombotic therapy and anticoagulation in fungal embolic disease is limited and there has been heterogeneity in observational data on their use in infective endocarditis comparing embolic events and fatal neurologic hemorrhagic complications [9-10]. Thrombolytic therapy is especially contraindicated due to the increased risk of intracranial hemorrhage [11-12]. Percutaneous coronary interventions, including balloon angioplasty and stent placement, have been described in similar cases [13-14]. These procedures increase the risk of distal embolization and dilation site infection. Stent placement particularly can increase the risk of mycotic aneurysm development due to intimal disruption and microbial seeding [15-16]. Therefore, aspiration thrombectomy appears to be a safer approach [17-20].

Our patient did not undergo a coronary angiogram and was medically managed for his myocardial infarction without anticoagulation, antithrombotic, or thrombolytic agent use. He underwent surgery with bioprosthetic aortic valve replacement and extensive debridement of the annular abscess with bovine pericardial patch placement. He has been following with cardiology and infectious disease outpatient clinics and was readmitted with fever and found to have multiple saccular aneurysms of the hepatic, superior mesenteric and left femoral artery, as well as a psoas abscess with a failed attempt at drainage by interventional radiology. He is maintained on life-long fluconazole, as he continued to have positive 1-3 ß-D-glucan levels without episodes of breakthrough candidemia.

A repeat transesophageal echocardiogram performed 10 months after the initial episode ruled out recurrent endocarditis. The patient has since developed ventricular dyssynchrony and was upgraded to a biventricular implantable cardioverter-defibrillator. Unfortunately, despite social and family support, he continued to use intravenous drugs.

## Conclusions

Septic coronary artery embolization from Candida endocarditis is an extremely rare phenomenon that is associated with poor clinical outcomes. Treatment involves intravenous followed by long-term suppressive oral anti-fungal therapy. Antithrombotic therapy and anticoagulation use in this scenario is controversial while thrombolytic therapy is contraindicated. The role of medical therapy and interventional revascularization procedures, including aspiration, has not been clearly established and needs further investigation.
